# State-of-the-Art Review on Wearable Obstacle Detection Systems Developed for Assistive Technologies and Footwear

**DOI:** 10.3390/s23052802

**Published:** 2023-03-03

**Authors:** Anna M. Joseph, Azadeh Kian, Rezaul Begg

**Affiliations:** Institute for Health and Sport, Victoria University, Melbourne, VIC 3000, Australia

**Keywords:** obstacle detection, wearable sensors, smart shoes, assistive devices, gait biomechanics, fall prevention

## Abstract

Walking independently is essential to maintaining our quality of life but safe locomotion depends on perceiving hazards in the everyday environment. To address this problem, there is an increasing focus on developing assistive technologies that can alert the user to the risk destabilizing foot contact with either the ground or obstacles, leading to a fall. Shoe-mounted sensor systems designed to monitor foot-obstacle interaction are being employed to identify tripping risk and provide corrective feedback. Advances in smart wearable technologies, integrating motion sensors with machine learning algorithms, has led to developments in shoe-mounted obstacle detection. The focus of this review is gait-assisting wearable sensors and hazard detection for pedestrians. This literature represents a research front that is critically important in paving the way towards practical, low-cost, wearable devices that can make walking safer and reduce the increasing financial and human costs of fall injuries.

## 1. Introduction

Extensive epidemiological and medical research now shows that it is imperative to prevent falls in older people and across a broad range of gait-impaired populations. The World Health Organization (WHO) identified falling as the second highest cause of unintentional death, after road accidents and approximately 37.3 million falls requiring medical attention occur each year [1]. In Australia, the cost of treating injury is the third highest category of healthcare spending after musculoskeletal disorders and cardiovascular diseases and annually AUD 3.6 billion is spent by the Australian healthcare system on fall-related physical injuries [2]. Many falls do not cause serious injuries, but one out of every five falls cause significant injury, with annually approximately 3 million older people treated in emergency departments due to a fall [3,4]. WHO statistics show that globally at least 2.2 billion people have a visual impairment, which in the case of older adults contributes to social isolation and walking difficulties, increasing their risk of falling and consequent likelihood of entering residential care [5]. Fear of falling also affects an individual’s quality of life by restricting everyday mobility and decreasing opportunities for recreation and social connection [6].

Biomechanically, falls result from any balance perturbations that cannot be restored [7] but tripping over obstacles is the principal cause of balance loss, accounting for more than 53% of falls [8]. Tripping can be defined as forceful, unanticipated contact with obstacles or the irregularities in the walking surface and reduced foot-ground clearance during the mid-swing phase of a gait cycle is highly hazardous [9]. The ability to continuously adapt foot trajectory to clear obstacles, such as roadside curbs or steps is critical to safe locomotion. In order to detect environmental hazards in the path of the user, obstacle detection technologies that perceive the environment, utilising sensors such as ultrasound, camera, infrared, radar, and laser range finder, have been extensively investigated in different domains [10,11,12,13]. Effective sensor integration with computational vision and environmental scanning is facilitating developments in real-time motion monitoring and effective interventions for fall prevention [14].

As the interface between the ground and foot, research in shoe-integrated technology began more than three decades ago, incorporating comfort and convenience into the design [15]. Technological transformations to the ‘smart shoe’ began with variables associated with walking speed and calorie consumption [16,17], advancing to rehabilitation applications [18]. Progress in wearable sensors, microfabrication, data acquisition, and processing combined with low-power, portable, wireless systems led to assistive footwear designed for individuals with visual or physical impairments.

Previous reviews have summarized the application and functionality of assistive devices, including shoe-based systems. Gokalgandhi, Kamdar [19] provided a review of smart technologies embedded in shoes, including electronic, mechanical, and electromechanical devices. Hegde, Bries [20] also summarized advances in footwear-based wearable systems, with applications in gait monitoring, plantar pressure measurement, posture and activity classification, body weight and energy expenditure estimation, biofeedback, navigation, and fall risk. Assistive technologies developed for visually impaired users have also been described [13,21,22]. Our aim here was to outline and critically evaluate shoe-integrated systems that incorporate obstacle detection to identify environmental hazards that pose a tripping risk. Despite technological advancements in assistive devices for collision avoidance in visually impaired individuals, the long cane and guide dog remain most commonly used. The white cane is preferred due to its reliability, simplicity, low cost, and minimal maintenance [22] but it does not entirely protect against collisions and guide dogs only provide support on familiar routes. There have, therefore, been developments in smart sensor-incorporated adaptations to assist navigation, including electronic canes/sticks [23,24], glasses [25], belts [26], caps [27], bracelets [28], and gloves [29]. Despite these advances, designers have not adapted them to the user’s gait characteristics [30] and thus people with navigational difficulties still remain less active.

### Obstacle Detection Smart Shoe System

**Figure 1:**
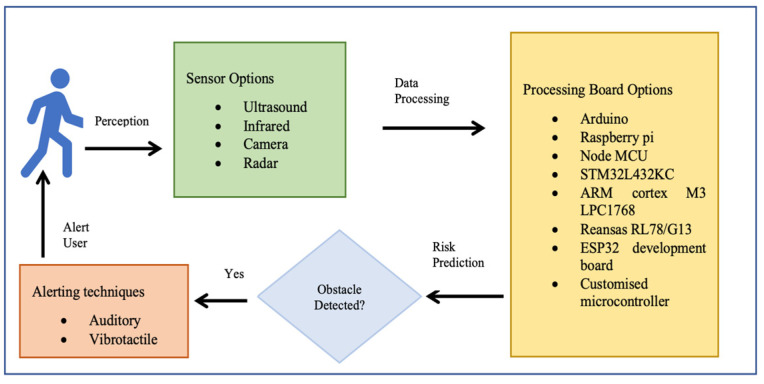
Schematic block diagram illustrating the main components of obstacle detection shoes, as used in the reviewed literature.

As shown in Figure 1 the fundamental requirements for an obstacle detection smart shoe are sensing, processing, and alerting. The sensing system has active or passive sensors to detect obstacles, while the processing unit for portable devices includes a microcontroller to trigger the sensor for perceiving, processing the data and analysing the risk of object contact. When an obstacle is detected the alerting system triggers an auditory or vibrotactile stimulus for an avoiding action. Selection of the hardware for each of these three principal units determines the effectiveness, reliability, and acceptance of the shoe system. Selected state-of-the-art shoe obstacle-detection systems are shown in Table 1 for illustration.

## 2. Sensor Technology in Obstacle Detection Systems

Obstacle detection and avoidance systems are widely prevalent in robotics and autonomous vehicles [39,40,41], to detect hazards in the environment. Sensors form an integral part of an obstacle detection system by perceiving the surroundings and converting that information into real-time data for further processing. Sensors can be classified as active or passive. Active sensors emit a signal and receive a distorted copy of the same signal, while passive sensors pickup an external signal to provide a corresponding output [42]. Active sensors for obstacle detection include radar (radio detection and ranging), lidar (light imaging detection and ranging), and sonar (sound navigation and ranging). Cameras that mimic the human eye(vision), are the most commonly used passive sensor. Table 2 provides an overview of sensors commonly employed in obstacle detection, and their characteristics.

Ultrasonic sensors, emitting high frequency sound waves above the range of human hearing, measure the distance to an object using a time-of-flight (TOF) technique [45,46], but they are limited by low angular resolution and cannot detect obstacle dimension. Ultrasound sensors can provide accurate short-range obstacle distance measurements, by emitting high-frequency (40 kHz) sound waves as a conical beam and detecting the reflected pulses [47]. Ultrasound sensors are widely used for obstacle detection because they are also compact and easily implemented in wearable assistive devices. To obtain a more complete picture of the environment, multiple sensors may be required and they can be confused by environmental noise and specular reflections [40].

Radar sensors emit high-frequency electromagnetic radio waves and typically adopt frequency-modulated continuous wave (FMCW) technology to estimate the target distance using the round-trip time principle, i.e., measuring the frequency shift between emitted and reflected signals [12]. Three types of radars employed in automotive systems are: long-range radar (LRR) for cruise control and collision avoidance, medium-range radar (MRR) for blind spot detection, and short-range radars (SRR) for parking assistance and proximity detection [10]. Long-range radars measure the vehicle’s speed and obstacles up to 200 m away using a 77 Ghz microwave radar but with low resolution. Short/medium range radars in the 24 Ghz and 76 Ghz bands also measure velocity and distance, but with limited resolution and complex return signals [10]. Despite limitations, radar sensors are employed very effectively in autonomous systems due to their reliable, accurate performance both day and night and in adverse weather conditions [48].

Lidar sensors have also been adopted in autonomous driving applications. They utilise shorter wavelength light sources giving high resolution, a wide field of view and fast sweep frequency [40,49,50]. Lidar sensors can be categorized as 1D, 2D, and 3D, differing based on the number of laser beams used and whether it is a point beam or scanning beam. Whereas 1D is range-only, 2D lidar has a single beam and spin, enabling 360° views and *x* and *y* axes data, the 3D lidar has several beams and 360° spin, providing object’s *x*, *y*, and *z* coordinates [51]. With high accuracy and long-distance measurement, 3D Lidars play a central role in obstacle detection for autonomous vehicles [52]. The point cloud obtained from Lidar has high processing requirements, is heavy and expensive, there is, therefore, work in progress to reconstruct a 3D environment, using 2D Lidar, reducing processing requirement and cost [53]. Lidars can also be either mechanical Lidar or solid-state (SSL), with mechanical version the most popular, using high-grade optics and rotary encoders driven by electric motors to capture 360° field of view. The SSLs uses micro-structured waveguides to direct the laser beams, eliminating the rotating lenses and reducing mechanical failures [48]. Even though the SSL has a narrower field of view than mechanical Lidars, typically 120° or less, they have gained interest in recent years due to being robust, reliable, and less expensive [54]. The higher frequency (10–20 Hz) and shorter wavelength of Lidar enable a more accurate measurement compared to radar sensors, with a typical accuracy of 1.5–10 cm, vertical angular resolution of 0.35–2 degrees, and horizontal angular resolution of 0.2 degrees [54].

Low-cost vision-based sensors such as cameras have been developed as the primary sensor for high-resolution obstacle detection. A camera detects the light emitted from the surroundings on a photosensitive surface, and redirects it through a lens, producing clear images [48]. Being relatively inexpensive, widely available, and providing rich contextual information similar to human vision, several obstacle detection applications have been developed using vision sensors. Passive cameras do not emit signals that may cause interference [42] and information in the form of pixel intensities captured with high-definition videos or images, can be used to extract shape, colour, and texture information, providing considerable environmental detail [48,55]. The considerable computational power demands of data processing is, however, a constraint, with the latest high-definition cameras processing multi-megabytes of real-time data [10] and they are susceptible to ambient light and weather conditions, with low illumination giving low quality images. Conventional monochrome cameras lack depth information, required for accurate size and position estimation of obstacles [42,55], although some applications can calculate depth information using complex algorithms [56]. A stereo camera with two image sensors can imitate 3D depth perception using epipolar geometry and triangulation methods [48] but demands more processing power. Time-of-flight cameras use an active sensor to measure the time taken for an infrared light beam to reach the object and reflect back to the camera, giving pixel depth and intensity, but distance accuracy and image quality are relatively low compared to other 3D sensors [57]. Microsoft Kinect is a popular range camera, capturing images and depth at high frame rates using a combination of RGB camera and infrared sensor. It has applications in mobile robotic mapping, navigation and localization, industrial robot collision avoidance, and human motion tracking [57], but it is too large for most wearable systems.

Infrared (IR) sensors are also active and passive in design, the active sensors emit infrared light to detect the reflections from the obstacles, the passive IR sensors detect the changes in infrared radiation striking them and are used primarily for motion detection [42]. There are advantages and limitations associated with each sensor type and when considering the applications to smart shoe systems, the size, weight, range, and real-time performance are also important. Table 3 gives the detailed specifications for a common sensor in each category taken from the literature and datasheets.

### Sensor Fusion

A single sensor may not provide sufficient information to measure the exact shoe-object distance and object size (height and width). Combining the information from multiple sensors, known as sensor fusion, provides a more veridical pictures of the environment, and this technique has also been demonstrated to be effective for obstacle detection in autonomous cars and mobile robots [44,70,71,72,73]. Integrating the acquired data from multiple modalities reduces the detection uncertainties and overcome the shortcoming of individual sensors operating independently [48]. A highly effective approach is to combine information from different types of sensors. In general, a passive sensor such as a camera, cloning human vision, will give richer information regarding the obstacle features and appearance, while active sensors e.g., ultrasound, lidar, and radar will be more accurate in estimating obstacle distance. Shahdib, Bhuiyan [74] used both ultrasonic sensor and a Canon 550 DSLR camera to guide an autonomous mobile robot, detecting obstacles and estimating their distance and dimensions. The advantages of data integration from multiple sensors are summarized below:

- Information obtained through fusion has a richer semantic and higher resolution than from a single sensor measurement.
- Joint information from multiple sources reduces the ambiguity and uncertainty with the measured values.
- Comprehensive coverage from multiple sensors gives extended spatial and temporal coverage.
- Increased confidence due to the availability of redundant information from many sensors scanning the same environment, and improved reliability due to sufficient information even if there is a partial failure.
- Reduce noise and errors through the fusion of multiple data, thus improving the accuracy [75,76].

The advantages of sensor fusion make it the optimal choice for a complex system, but the number and the type of sensor must consider their cost, size, and system’s application. In addition, the factors considered for a wearable system are different to those in autonomous cars and robots. Considerations for shoe-integrated obstacle detection are whether sensors can provide environmental data for safe navigation but also be compact, lightweight, and portable. In addition, fast, reliable data processing is required to alert the user of hazards in their walking path. The following section examines sensors used for obstacle detection while walking.

## 3. Walking with Obstacle Detection

**Figure 2:**
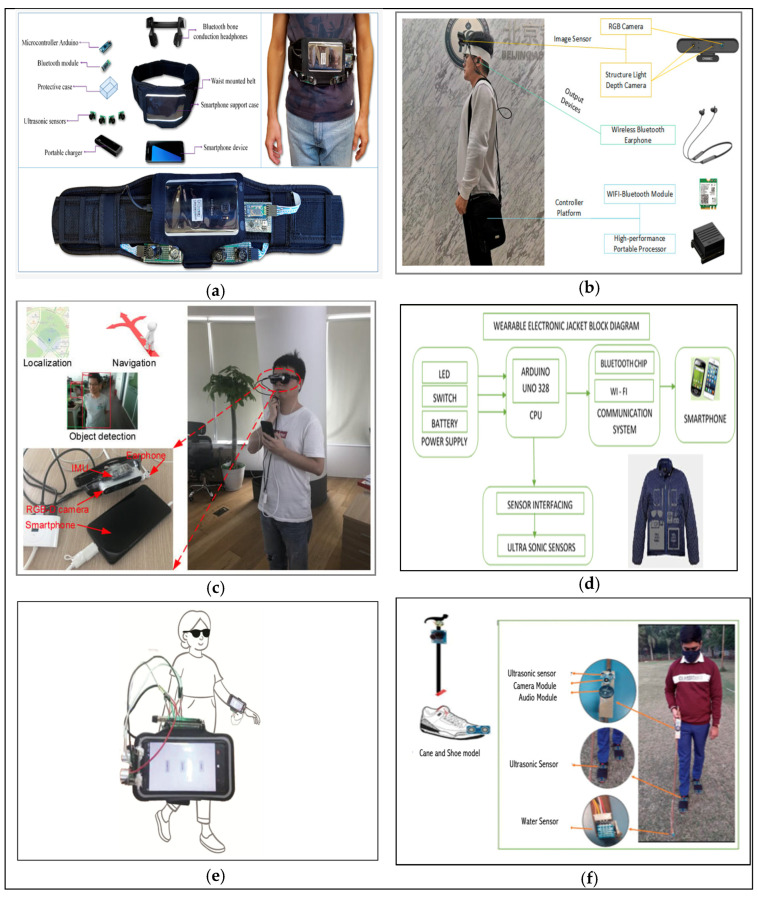
Illustration of some wearable assistive technologies with obstacle detection incorporated. (**a**) Smart Belt [87]. (**b**) Head-Mounted [88]. (**c**) Smart Glass [89]. (**d**) Smart Jacket Reprinted with permission from Ref [90].Copyright 2018, Elsevier. (**e**) Wrist-worn navigation device [22]. (**f**) Vision Navigator with Smart-fold Cane and Smart-alert walker [91].

Walking safely through the everyday environment is essential to healthy, productive lives and developments in gait assisting technologies incorporating wearable sensors are progressing rapidly [21]. Assistive technology encompasses devices, services, systems, and environmental modifications to enable individuals with locomotor impairments to overcome barriers to independence [77]. Research into the navigational aids and obstacle detection systems in assistive technologies is quite extensive. Portable assistive devices can be moved from place to place providing safer navigation for independent living and rehabilitation. Wearable assistive devices can be attached to wristbands [78], eyeglasses [79,80], head-mounted devices [81], vests [82], belts [83], shoes [34], and any other practical point of attachment, allowing hands-free interaction. Smartphones provide portability and convenience and have become a core assistive tool in supporting navigation by obtaining information and interacting with the user’s environment [84]. Microsoft Kinect, initially developed for gaming, has become popular among vision researchers and assistive technologies due to the cost, detection capability, and data acquisition software [85]. With the miniaturization of the electronics devices and advancements in computer vision and machine learning algorithms, research in wearable navigation devices have also incorporated these techniques and sensor fusion methods [86]. Figure 2 illustrates some of the wearable assistive devices developed for a safe navigation of the visually impaired. Some commonly used obstacle detection sensors in wearable assistive device and their specifications and feedback mechanisms are presented in Table 4.

Despite progress in the design of obstacle detection devices their acceptance by users in daily life is limited. Many navigation systems have been proposed for the visually impaired but few allow successful dynamic interactions and adaptability to changes and none can work seamlessly indoors and outdoors [97]. Poor user interface, functional complexity, weight, size, and cost have been identified as contributing to the low acceptance of electronic travel aids [31]. For these systems to meet user requirements, clear and precise object detection from close proximity to a minimum of 3 m are necessary. The section below outlines developments in shoe integrated assistive systems.

## 4. Obstacle Detection in Shoe-Based Assistive Devices

Incorporating technological features into a shoe avoids having an additional item to be worn or carried and with optimal design can be lightweight, affordable and comfortable. In addition to obstacle detection, smart shoe systems have incorporated features such as live location tracking, heat sensing, slippery surface detection, fall detection, electricity generation while walking for an alternate power source, health and fitness tracking, and pothole detection [33,98,99]. Table 5 highlights the main features of smart shoes with obstacle-detection technology, collected from the reviewed papers.

The most frequently targeted application for obstacle detection in smart shoes is visual impairment [30,31,33,34,35,36,37,38,91,98,99,100,101,102,103,104,105,108], with less work on obstacle detection for the physically challenged, where the gait impairments must also be considered. With the elderly population expected to almost double from 15% of the total population in 2015 to 22% by 2050 [109], there is a need for continuing research into devices that can detect hazards and prevent falls in individuals with walking impairments.

Smart shoe systems designed primarily for obstacle detection may have additional features, such as communication with a smartphone allowing location tracking and contacting someone for emergency assistance [34]. As well as smart shoes for obstacle detection, the Smart Bottine, designed to help individuals with autism, incorporates an android smartphone with the Blynk application for notifying the caregiver in case of emergency [32]. Additional features to provide support for the visually impaired include location tracking service using GPS-GSM, android application for locating missing persons and emergency calls(SOS) with location tracking using Google Maps [33]. Navigational guidance has also been facilitated with a Bluetooth transceiver mounted on the shoes, synchronized to a smartphone application using Google Maps [35]. COMPASS [105], an indoor position system, is targeted towards visually impaired university students, with the smart shoes detecting obstacles and an additional smart bracelet with a camera to verify the correct classroom.

Smart shoe systems can work well on their own [30,31,33,34,35,36,37,38,91,98,99,100,101,102,104,105,106,107,108], but some researchers have used it in conjunction with other obstacle-detection devices [91,103]. Chava, Srinivas [103] incorporated smart glass with smart shoes, with sensors attached to the spectacles to detect head level objects and integrated to Bluetooth hearing device for voice commands. ‘Vision Navigator’ [91] was designed as an assistive interface for users with low or fluctuating vision for indoor and outdoor navigation. The Smart alert walker consists of sensor-equipped sneakers with two built-in ultrasonic sensors to identify short-range obstacles. It is used only as an emergency backup with the Smart-fold cane as the primary system for detecting obstacles and containing all the major hardware components. The intelligent obstacle detection model was deployed for the image obtained from the camera-in-cane, using the SSD-RNN (single shot detection- recurrent neural network) approach, computing with an optimum accuracy of 95.06% and 87.68% indoors and outdoors, respectively [91]. A considerably different approach uses a thin, flexible metal wire antenna running along the shoelace for collision avoidance in the front, but without feedback to alert the user [110].

### 4.1. Sensors in Obsctale Detection Shoes

**Figure 3:**
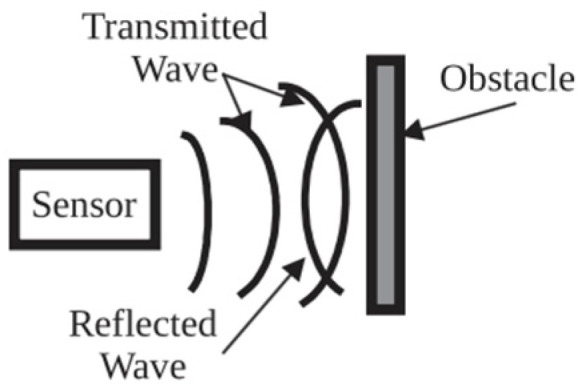
Obstacle detection based on reflection of a sound wave from an ultrasonic sensor. Reprinted with permission from Ref. [31]. Copyright 2018, IEEE.

Of 20 reviewed papers 17 reported using high-frequency ultrasound sensors utilizing echolocation principle to transmit and receive sound waves for detecting and locating obstacles [32,33,34,35,36,91,98,99,100,101,102,103,104,105,108]. Objects must be either directly in front or at a minimum angle to the transmitter, to be reflected and received by the ultrasonic receiver [31], as shown in Figure 3. Accurate ultrasound detection of up to 4 m allows the detection of the obstacles accurately, and to avoid false detection in crowdy scenarios, a customizable mode with a range of 0 to 1 m has also been deployed [108]. With a wide field-of-view ultrasound sensors are efficient in detecting the distance to an obstacle but lack the ability to accurately determine the direction of the obstacle.

Rather than mounting a single ultrasound sensor on the shoe, multiple units can alert to obstacle in the direction of attachment [37,103], due to small size, low cost, and high reliability [98]. NavGuide [31] uses six ultrasonic sensors, to identify obstacles in their respective scanning fields and map the position of each obstacle in front, on both sides of the shoe, detecting floor-level obstacles, knee-level obstacles, and the risers of an ascending staircase [31].

**Figure 4:**
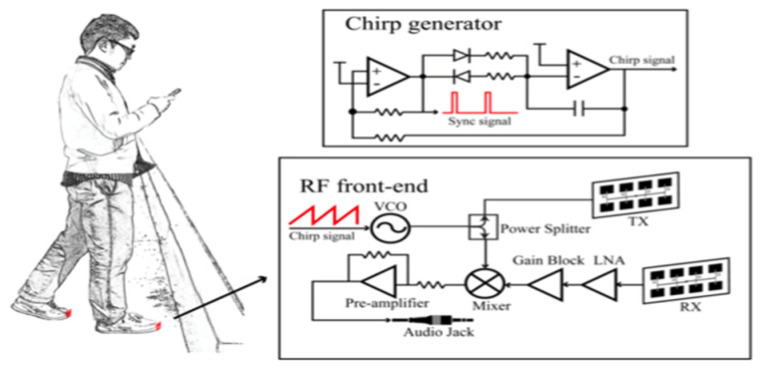
Using an FMCW radar for fall prevention. Reprinted with permission from Ref. [107]. Copyright 2016, IEEE.

While most shoe-based systems have utilised ultrasound, the feasibility of wearable radar sensors for detecting non-conductive obstacles and floor/wall surfaces has been demonstrated using a 60 GHz System-on-chip mm-wave radar, and a Texas Instruments IWR684ISK and MMWAVEICBOOST, connected to a laptop via USB port [111]. A novel frequency-modulated continuous wave (FMCW) radar transmits a linear chirp signal and calculates object distance based on round-trip time delay. The shoe prototype consists of two wearable K-band radars mounted on shoes (see Figure 4) to detect the absolute distance to an object and the shoe-ground clearance [107]. In another approach, Yang, Jung [30] utilised direction controlled infrared sensors with a narrow detection range to distinguish the object direction.

**Figure 5:**
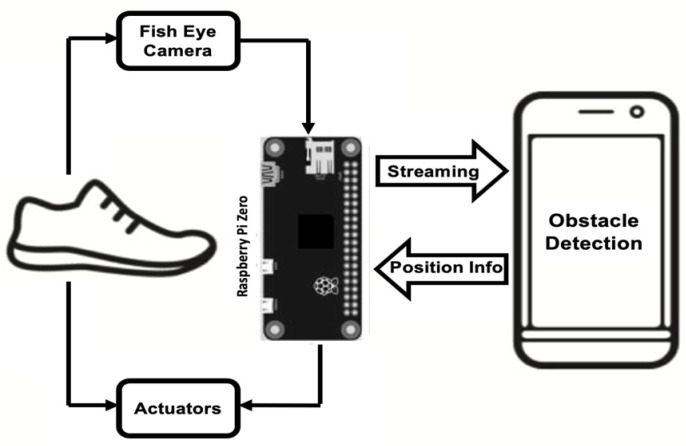
Block Diagram of the Smart shoe system with the fisheye camera. Adapted with permission from Ref [36]. Copyright 2021, IEEE.

Lin, Yang [112], proposed a camera-based line-laser obstacle detection system, using a Logitech C310 webcam operating at 29 frames per second with 640 × 480 resolution, and a 405 nm wavelength laser. A fall prevention strategy was implemented with a Sum of Absolute Difference threshold to trigger the obstacle detection event, line-laser pattern segmentation, homography transformation, and obstacle danger-level, showing the possibility for installation on shoes. Staircases, potholes, and ditches can also be predicted using a Yolov3 model from the video output of a simple 5MP fish eye camera with a 120-degree field of view [36], as illustrated in Figure 5.

While a camera can provide reliable obstacle information, the need for a high-performing processor remains a problem when deployed as a vision sensor [30]. The implementation practicalities and accuracy of compact cameras for image capture in other assistive devices [91,103], shows potential for smart shoe applications. The SSD-RNN model, based on the input from the camera deployed in the walking cane system described earlier gave optimum performance, generating 95.54% recognition accuracy for common obstacles [91]. Compact, lightweight cameras, such as the Raspberry Pi camera, and high-performing microcontrollers could be deployed in the shoe systems, avoiding the requirement for a walking cane. Many other sensors also have potential for implementation in shoes, outlined below.

### 4.2. Additional Sensing Deployed

Along with obstacle detection, additional sensing methods are also deployed in the obstacle detection system, considering further safety features.

- Pothole Detection: Potholes can be detected with infrared sensors [33,99].
- Water Detection: When the surface of a water sensor comes into contact with water, it returns a non-zero value, indicating any water source including wet floors [31,33,37,91]. Soil dampness sensors measure the volumetric water content in soil [33,113].
- Heat Sensing: The LM35 temperature sensor detects fire or a hot object near the user and the information can be relayed using voice commands [33].
- Insect Detection: The movement of insects/reptiles in a shoe triggers the infrared sensors in the shoe, notifying the user via the Blynk app in a smartphone or through email [32]. This insect detection is activated when the user is not using the shoes.
- Fall Detection: Foot motion data detected with motion sensors can be used to recognise a fall and alert an emergency contact via smartphone [32,34].
- Location Tracking: Whether the location is tracked for safety or when a person is missing, the use of smartphones ensures reliability and portability. Google’s Geolocation API makes use of Wi-Fi to determine the coordinates of the device and with a request to Google Maps, the user’s location can be mapped [32]. Bhongade, Girhay [33] reported on a system that tracks the location by sending a SMS code to receive the coordinates and navigate to the location using Google Maps.
- Height Detection: The height of the distant object in the frontal plane can be detected with a sensor integrated knee band [101].
- Text Detection: Verification of the classroom number utilizing a raspberry pi with an 8 MP Sony IMX219 image sensor to capture the images of the classroom tags and covert it to the desired form using OpenCV and Tesseract [105].
- Gait Detection: To detect the gait phases, based on which the obstacle detection could be triggered, motion data from an accelerometer [34] and the difference between two successive frames of a camera [112], were utilized.
- Health Tracking: Daily activity such as the number of footsteps, distance travelled and burned calories can be recorded for one week and accessed by the user [33].

### 4.3. Microcontroller Unit

A microcontroller unit is the core of a wireless obstacle detection system, consisting of a processing unit, memory and input/output peripherals. It receives an input signal from the sensor(s), process the input data, estimate the target parameters, and makes a prediction. The desired features such as low cost, power efficiency, and small size, make microcontrollers with arm processors a suitable option for a wide number of portable applications. Table 6 shows the technical features of microcontrollers used in the reviewed obstacle detection shoes, when unreported the principal features were obtained from the corresponding datasheets issued by the manufacturers.

Other high-performing microcontroller boards in the market such as Google Coral Board [118], Raspberry pi 4B [119], and Jetson Nano [120], with specifications showing the possibility of implementing in obstacle detection shoes with high performing sensors and machine learning algorithms for reliable and accurate fall prevention systems. Google Coral development board [118] is a single board computer that has an NXP i.MX 8M SoC processor based on Arm Cortex-A53 and an Edge TPU co-processor, providing accelerated machine learning processing. It includes all peripheral connections such as USB 2.0/3.0 ports, DSI display interface, CSI-2 camera interface, Ethernet port, speaker terminals, and a 40-pin GPIO header, all useful in developing a prototype smart shoe system. The NXP’s iMX8M system-on-chip (SOC) and the Edge TPU coprocessor together with LPDDR4 memory, eMMC storage, and dual-band Wi-Fi, form a removable system-on-module (SOM), enabling prototype development on the Google Coral development board and then combining SOM with a custom baseboard. The Google Dev board mini [121] also provides fast machine learning (ML) inferencing in a small form factor. The Coral USB accelerator [122] can be added as an Edge TPU co-processor to a computer system to perform accelerated ML inferencing. It is capable of performing 4 trillion operations per second, with 2 watts of power, USB 3.0, enabling on-device machine learning processing.

While the specification of the google coral devices shows the possibility of implementing machine learning algorithms in embedded devices, the support and guide provided by Raspberry pi make it easier to use and more convenient. The latest Raspberry pi 4 Model B [119] includes a high-performance 64-bit quad-core processor, dual-display support at resolutions up to 4K via a pair of micro-HDMI ports, up to 8GB of RAM, dual-band 2.4/5.0 GHz wireless LAN, Bluetooth 5.0, Gigabit Ethernet, USB 3.0. For additional performance, it can also be used with the Coral USB accelerator. The Jetson nano Developer Kit [120], is a small powerful computer, with 64-bit Quad-core ARM A57 @ 1.43GHz, 128-core NVIDIA Maxwell @ 921MHz, 4GB 64-bit LPDDR4, four high-speed USB 3.0 ports, MIPI CSI-2 camera connector, HDMI 2.0 and DisplayPort 1.3, Gigabit Ethernet, M.2 Key-E module, MicroSD card slot, and 40-pin GPIO header capable of running multiple neural networks in parallel for applications such as image classification, object detection, segmentation, and speech processing.

Researchers have evaluated these hardware developments for their capability in embedded object detection [123,124,125,126]. Real-time implementations of edge-based obstacle detection models in advanced processors for robotics [127,128], autonomous vehicles [129,130], wheels chairs [131,132], and visually impaired [133,134,135] calls for investigations of the same processors for shoe-based detection systems. Even with these high specifications, when selecting a microcontroller for a wearable system, along with the performance, the size, and weight are also important determinants of acceptance by a user.

### 4.4. Feedback/Alerting Technique

Acoustic and vibrotactile feedback are the most common methods to alert the user to obstacles. Acoustic feedback uses sound to capture the user’s attention, while in tactile or haptic feedback, embedded vibrators use the pressure on the skin. Auditory warnings can be a tone, buzzer or audio messages. Piezo buzzers are output devices, containing piezo crystals that expand and contract proportional to the applied voltage, producing sounds to alert the user [32,37]. Based on the proximity signal intensity can be controlled using pulse width modulation to produce a louder noise for closer obstacles [38]. Audio messages are either synthesized or digitized. Bhongade, Girhay [33] used an android text-to-speech application to alert the user to obstacles and provide date and time via headphones. A difficulty with audio outputs is interference with environmental information and they may be aversive for some individuals.

Vibrotactile warning mechanism, such as used in smartphone alerts, can be embedded insole or on the shoe, and sometimes combined with actuators. A signal enabling the vibration to alert the user is sent from the microcontroller when an obstacle is detected [34]. A coin vibrator alerts with a vibration amplitude proportional to the distance to the obstacle [98]. Feedback of obstacle direction can be achieved using four vibrators, i.e., each for right, left, and forward and all four to signal stopping [103].

With the implementation of features additional to obstacle detection, other feedback mechanisms have been employed. NavGuide [31] alerts with audio and tactile output, with the audio feedback playing an audio file corresponding to the detection and tactile feedback involving vibration motors front, left, and right corresponding to the direction and the fourth for wet surface detection. Smartphone-based voice guidance and vibrational feedback are implemented with vibrations motors in the both shoe insoles alerting the user to potholes and pedestrians with their directions and simultaneous activation of both indicating staircase detection [36]. Appropriate choice of feedback method affects the implementation of the system in real world. While incorporating all feedback methods in a single system might not be fruitful, restricting with one feedback method, can be also challenging in some situations, for example, audio feedback might not be suitable for noisy environments but preferred in other situations [21].

### 4.5. Analysis of Obstacle Detection Techniques

Ultrasound being the most common obstacle detection sensor used, time-of-flight technique have been utilized to sense obstacles in the path [32,33,34,35,36,91,98,99,100,101,102,103,104,105,108]. A microcontroller triggers the ultrasound to emit waves at short intervals and the obstacles reflect these waves back to the sensors [31]. The object distance was computed from the period between emission and reception of ultrasound waves, *D =* ½ *C* × *T*, where *C* is the speed of the sound in air and *T* is the measured time of flight taken by the sound wave [105]. Distance information is passed on to the processor, to alert the user about the presence of an obstacle. A comparison between the ultrasound measured distance and actual distance showed an accuracy of 98% which decreased with increase in distance, showing 94.78% at 300 cm [37].

Detection of obstacles at different levels was achieved with multiple ultrasound sensors and appropriate calculations. In NavGuide [31] a logical map of the surrounding environment was constructed with six ultrasonic sensors, divided into two groups-Group 1 (*S*1, *S*2, *S*3) for floor level obstacles and Group 2 (*S*4*, S*5*, S*6) for knee level obstacles. Front facing *S*1 and *S*4, left facing *S*2 and *S*5, and right facing *S*3 and *S*6 detect obstacles in the corresponding direction in which they are placed. Obstacle *x*-coordinate value was calculated from the measured distance for all sensors.*x_coordinate_ = cos θ × D_i_*. Here, *Di* is distance calculated by ith ultrasonic sensor (*i* = 1, 2,..., 6) and *θ* is angle of the sensor with the horizontal. The presence of knee level obstacles was determined with the below equations.

[(*S*1*x* < *S*4*x*)*&&*((*S*4*x* − *S*1*x*) ≤ *δ*)] (1)

[(*S*2*x* < *S*5*x*)*&&*((*S*5*x* − *S*2*x*) ≤ *δ*)] (2)

[(*S*3*x* < *S*6*x*)*&&*((*S*6*x* − *S*3*x*) ≤ *δ*)] (3)

where *S*1*x* represents *x*-coordinate value calculated by *S*1, *δ* is the width difference between *S*1 and *S*4 and γ the height difference.

Additionally, ascending staircase was detected in the front, left and right using the below equations.

[((*S*1*x* < *S*4*x*)*&&*((*S*4*x* − (*S*1*x* + *δ*)) ≥ *Td*))] (4)

[((*S*2*x* < *S*5*x*)*&&*((*S*5*x* − (*S*2*x* + *δ*)) ≥ *Td*))]. (5)

[((*S*3*x* < *S*6*x*)*&&*((*S*6*x* − (*S*3*x* + *δ*)) ≥ *Td*))]. (6)

*Td* represents tread depth (25 cm) and *Rh* is the value of the riser height (19.6 cm) [31].

Additional sensing techniques such as wet surface detection, pothole detection etc., running in parallel to obstacle detection and custom smartphone applications communicating with the smart shoe processor [32,33,35] extends capabilities of the shoe system. For COMPASS [105], in addition to, an ultrasound-based obstacle detection, a computer vision coupled with indoor position system solution was used for the independent navigation of the visually impaired students in university campus. An android application reminded the student on class timings while also providing navigational assistance. Shoe-mounted sensors detected obstacles and Bluetooth beacons tracked user’s location. At destination, for verifying the classroom number, image captured with a camera on smart bracelet is converted to text using OpenCV and Tesseract. After text-to-voice conversion with Google library, the audio output confirms the classroom number to user [105].

Vision navigator [91] uses the ultrasound in the shoe system (Smart-alert walker) in conjunction with a Smart-fold cane. An Arduino embedded with a Raspberry Pi camera act as the heart of the system. These were attached to the Smart cane to take live camera feed and utilized a SSD algorithm trained with MS COCO dataset to detect the potential obstacles. This was validated with trained deep learning model and transferred to RNN for sentence generation. Appropriate sentences were framed by interacting with Flickr30k dataset, which was then forwarded to text-to-speech application interface for audio alert through earpiece. Two ultrasonic sensors in Smart-alert Walker served as emergency alert provision, by detecting any obstacles that are too close, enhancing the accuracy of the system [91].

Though ultrasound was used in most systems, two other sensors have also used the signal round trip travelling technique for obstacle distance. Yang, Jung [30] used infrared sensors and six-axis motion sensors mounted on shoe to estimate the obstacle distance and direction. Corresponding to the foot angle, the direction of infrared sensor was adapted, to detect and alert to presence of obstacles in corridor environment. Tang, Peng [107] proposed a radar-based fall prevention approach that constantly measures distance information between surrounding objects and user’s feet. While the measurement of radar sensors showed same spectrum characteristics in normal walking scenario, appearance of obstacle in the radar detection range was captured as fragmented distance decrease. Comparison of the distance results measured by a prototype K-band FMCW radar and the ground truth value showed 1.76 cm average error and 4.5 cm worst-case error.

Rao and Singh [36] implemented a computer vision approach for obstacle detection and avoidance, guiding the user with appropriate haptic feedback and navigational support with smartphone voice assistance. The image captured with a fisheye camera on the shoe was streamed to an android application in smartphone. Obstacle detection was performed with a YOLOv3 model, trained with Darknet suit, converted into TensorFlow lite file, and integrated into the smartphone application. The application detected potholes, ditches, staircases, and people to understand crowded places. The distance to the object was estimated with the principle of triangle similarity represented with F = P × D/W, where W is the actual width, P is the perceived width, and D is the distance from the camera. F remains constant and was calculated for some standard examples, from which the distance was estimated D = W × F/P. A haptic alert was initiated whenever the obstacle distance falls below certain threshold, informing the user next direction to move [36].

It is clear from the review that in shoe-based obstacle detection systems, there is a gap in using advanced sensing and detection techniques. The implementation of deep learning and machine learning algorithms in other wearable assistive devices [86,136], shows a scope for the same in shoe-based systems.

### 4.6. Communication

Communication between the sensors, microcontroller boards and other devices can be achieved via wired or wireless protocols. With advances in wireless protocols, microcontrollers are now available with in-built wireless systems, or separate wireless modules can be used. Bluetooth short-range wireless transmission between 2.4 GHz and 2.485 GHz enables low cost communication with minimal power consumption [137]. The HC-05 Bluetooth module is ideal for transferring real-time shoe data between the microcontroller and the smartphone [33,35,37] but they must be within a specified range to function effectively. Kamaruddin, Mahmood [102] controlled a buzzer from the microcontroller through the internet using Wi-Fi module. Using internet communications location information from the user can also be made available to supervising personnel [38]. Wireless communication between smartphones and smart shoes has, therefore, paved the way for safety interventions using shoe system, such as location tracking and navigation [32,33,35].

### 4.7. Power Supply

Hardware components including processors and sensors demand a portable power supply of sufficient duration. In NavGuide [31], with power consumption between 48 mA and 120 mA, and changing surroundings, a compact battery enabled 850–1000 min of operation [31]. A Li-ion rechargeable 12 volts battery with sleep mode consumption of less than 2 mA, can support sensors and other hardware components [35]. Daou, Chehade [98] used two batteries to power up their system, by switching from the main battery to the second, when the former battery level reached 10%. With a power consumption of 708.9 mA the average life cycle of a 600 mA, 9 V battery is about 40 min [98].

**Figure 6:**
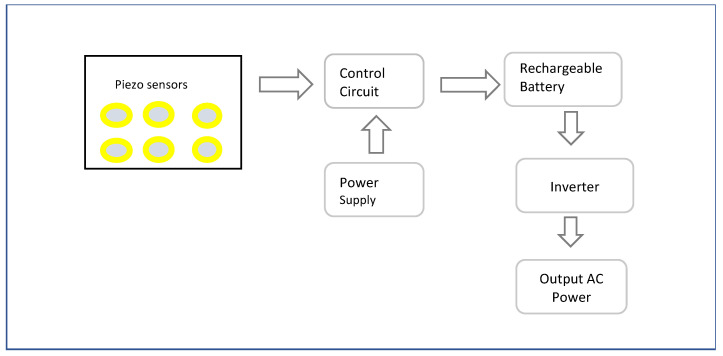
Flow of energy generation from Piezo electric plates. Adapted with permission from Ref. [138]. Copyright 2021, IEEE.

While a lithium polymer battery powers the system, piezoelectric plates in the shoe sole are used to generate and store backup power from foot pressure applied when walking and running [33] allowing the wearer to maintain power to the sensors and smart phone [113]. Piezoelectric sensors convert applied pressure into electrical energy, filter AC content to produce DC and store generated power in a rechargeable battery [138] (see Figure 6). For powering AC systems, an inverter can convert the stored energy, thus showing possibility to support both DC and AC loads. With a 100% capacity a rechargeable battery was observed to support the shoe system for 3–4 h, and piezoelectric plates produced addition power but less current [33]. The Walking energy module of a Smart shoes [34] consists of a MAX17710 energy harvesting charger and protector, collecting the electricity power from the Piezoelectric transducer with Polyvinylidene Fluoride (PVDF) thin film, under the heel. Energy harvesting from human locomotion promises to be a convenient way to power versatile smart shoe systems.

### 4.8. Experimental Evaluation with Human Participants

Performance of wearable shoe systems should be confirmed in trials with human participants to ensure effectiveness in detecting the obstacles. Daou, Chehade [98] tested a system with five participants, for an average duration of 2 h per user, obtaining an accuracy of 95.33% with a sensitivity of 98% and a false detection rate of 5.3%; most errors occurred when the battery level was low. In another study, the effectiveness of smart shoes in gait event recognition and obstacle detection was assessed using six participants, with the gait event showing an overall accuracy of 90.9% and low variability in real-time, with high detection accuracy and low false-alarm rate [34].

Two shoe systems have tested the shoe-based obstacle detection systems and compared it along with a white cane system, the most commonly used assistance for the visually impaired. For testing NavGuide [31] experiments were conducted in a controlled environment with 70 participants. Following a training trial, performance was measured using the number of obstacles contacted, time, and speed to complete walking task, and success in wet floor detection. Results showed that the NavGuide assistance reduced both collisions and completion time compared to the white cane. Yang, Jung [30] also compared the shoe performance with white cane walking in 12 participants. Their results generally supported the earlier test results, but time to pass increased using the obstacle detection footwear, while more collisions were avoided and plantar pressure distribution and muscle activity improved. In summary, limited research has experimentally evaluated the performance of the developed shoe systems. While bench testing can estimate reliability, it is critical to find out how the system performs in simulated and real-world environments.

## 5. Conclusions

This review has described recent developments in portable and wearable obstacle-detection shoe systems to detect hazards and to reduce accidents while walking. The sensors used for detecting the hazards were examined, with the major advantages and disadvantages of each, and a detailed analysis provided of the sensors and hardware components used in obstacle detection shoes. Wearable sensors have the potential to improve the quality of life for individuals with locomotor deficits and enabling new scientific concepts in hazard detection and gait monitoring. Audio or tactile stimuli are the most common feedback methods used to alert the user of hazards. Integration of additional safety features, for example, the detection of wet surfaces, potholes, and heat, in addition to location tracking and navigation, promise more secure and safe independent mobility.

Our review of shoe-based obstacle detection systems reveals that while research has progressed, more advanced motion prediction algorithms and techniques are required. Despite technological advancements in sensors suitable for obstacle detection, most smart shoe systems have used only ultrasound-based sensors, with major application in visual impairment. Adopting more advanced sensor technologies and data processing may help in designing more efficient diagnostic methods, leading to practical, cost-effective, technology-based fall prevention interventions.

To pave the way for high-quality, efficient, reliable obstacle detection on shoes in real time, bridging the gap between research and practice, we make the following recommendations:

- Design sensor systems that reliably detect obstacles using multiple data sources and only those that pose a hazard to the user, i.e., few false positives.
- Implementation of advanced wearable sensors and fast processing boards on the shoe while not compromising user comfort and ease of use.
- For prototype development, microcontrollers such as Arduino may be suitable but for real world applications smaller processors/boards with equivalent or advanced processing capabilities are needed.
- Ensure the additional hardware and weight, do not interfere with the gait and normal locomotion of the user.
- Examine temperature ranges for sensors (see Table 3) and other hardware components to determine the performance in extreme weather conditions.
- Evaluate prototypes in the real world to ensure comfort and acceptance for everyday use.

Application of properly developed smart shoes can be envisioned in any discipline promoting independence, convenience, individualized comfort, and healthy living. Innovative smart shoes have the potential to revolutionize the footwear industry and create a new interdisciplinary science of sensor technology, computing, and gait biomechanics.

## Figures and Tables

**Table 1:** Examples of smart shoe systems selected from the reviewed articles. Only the sensors used for obstacle detection by the shoe systems are listed in this table.

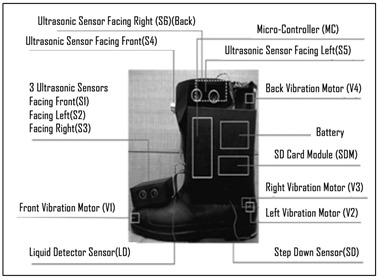 Patil, Jawadwala, 2018 [31]-(copyrights authorized by IEEE)	Obstacle Detection Sensor: Ultrasound Processing Board: Customized Microcontroller Feedback: Audio, Vibration Functionality: Detecting the floor-level up to knee-level obstacles in front, on the left, and on the right side of the shoe system Wet floor detection Tactile feedback through vibration motors and auditory feedback through user’s wired or wireless headphones
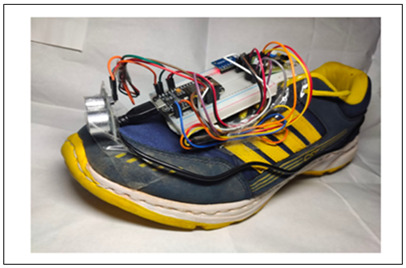 Nanduri, Umamaheswari, 2022 [32] (copyrights authorized by Elsevier)	Obstacle Detection Sensor: Ultrasound Processing Board: Node MCU Feedback: Smartphone, Piezo buzzer Functionality: Detect obstacles in the path and detect insects in the shoe while shoe is not in use Fall detection and notifying the parent/caretaker through smartphone Location tracking through Google’s Geolocation API
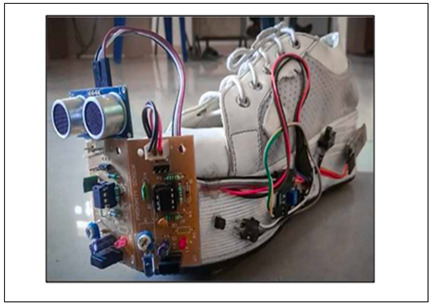 Bhongade, Girhay, 2022 [33]-(copyrights authorized by IEEE)	Obstacle Detection Sensor: Ultrasound Processing Board: Arduino Atmega328 Feedback: Audio Functionality: Obstacle detection to identify obstacles Navigation and location tracking Pothole detection, slippery surface detection and hot objects or fire detection Emergency SOS to family members Electricity generation while walking Health and Fitness tracking Alerting through voice instructions
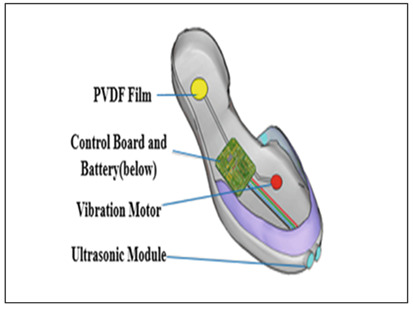 Wu, Lei, 2021 [34] (copyrights authorized by Springer nature)	Obstacle Detection Sensor: Ultrasound Processing Board: STM32L432KC Feedback: Vibration Functionality: Detect low obstacles and alert through vibration Gait events detection to detect motion state of the feet Fall detection and contacting emergency contacts through cell phone Recharge battery with electricity generated through walking
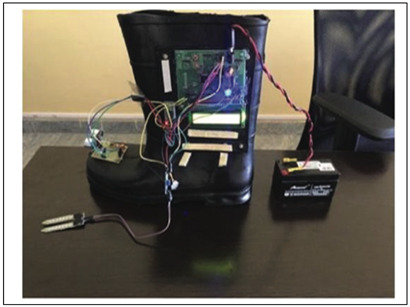 Pradeep Kumar, Inchara, 2021 [35]-(copyrights authorized by IEEE)	Obstacle Detection Sensor: Ultrasound Processing Board: Renesas microcontroller Feedback: Smartphone audio output Functionality: Obstacle detection and moisture detection Location notification using google maps User alert through audio feedback
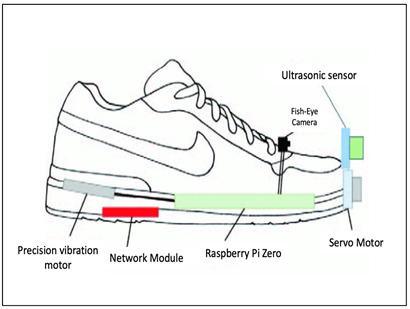 Rao, Singh, 2021 [36]-(copyrights authorized by IEEE)	Obstacle Detection Sensor: Ultrasound, Fisheye camera Processing Board: Raspberry Pi Zero Feedback: Vibration Functionality: Pothole and staircase detection, people detection to determine crowded places Vibration on the left and right to alert about detection on the corresponding side and vibration of both to alert about staircases Navigation using google map API integrated with voice-based interface for audio guidance
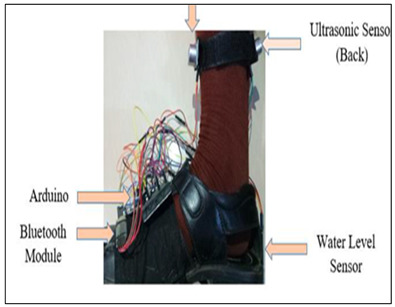 Rahman, Islam, 2019 [37]	Obstacle Detection Sensor: Ultrasound Processing Board: Arduino Uno Feedback: Buzzer, Smartphone Functionality: Detect obstacles in the front and backside Wet or slippery surface detection Feedback through buzzer and smartphone notification as audio through earphone
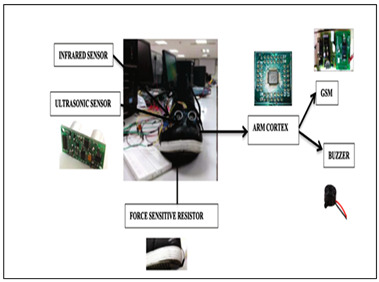 Raja and Santhosh, 2021 [38] (copyrights authorized by Elsevier)	Obstacle Detection Sensor: Ultrasound, Infrared Processing Board: ARM cortex M3 LPC1768 Feedback: Buzzer Functionality: Obstacle detection and false positive detection Detect whether the shoe is worn or not Varying sound intensity based on the distance to obstacle Distance parameters of user send to caretaker through cloud, using WI-FI module
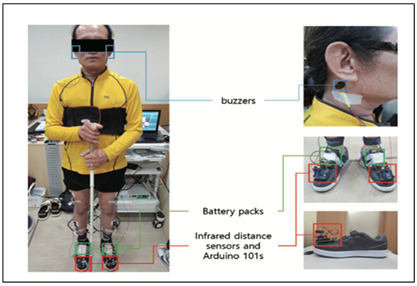 Yang, Jung, 2020 [30]	Obstacle Detection Sensor: Infrared Processing Board: Arduino Feedback: Piezoelectric Buzzer Functionality: Detect obstacle and direction of the shoes Direction control of the infrared sensor using the data from accelerometer and gyrometer Buzzer alert for obstacles within 60 cm

**Table 2:** A Summary table of Sensor Features [40,42,43,44].

SENSOR	PERCEIVED ENERGY	ADVANTAGES	DISADVANTAGES
LIDAR (3D)	Laser Signal [emitted]	• Accurate distance measurement • Wide field of view • Precise measurement of depth • 360° high-resolution mapping • Can measure outlines of objects • Unaffected by lighting conditions	• Expensive • Affected by dust, rain, and snowy conditions • Only objects in the scanning plane are detected • 3D point cloud storage requires large memory • The point cloud is sparse
RADAR	Millimeter wave radio waves [emitted]	• Reliable • Accurate distance and relative speed measurement • Suitable for medium to long distance range (200 m) • 150° wide field of view • Good Angular Resolution • Robust in different weather and environmental conditions	• Expensive • Heterogenous reflectivity of materials makes processing tricky • Lower processing speed compared to camera and lidar • Lacks fine resolution needed for obstacle detection
Camera	Visible light [ambient]	• Low cost, Compact size • Rich Contextual information • Vision similar to human eyes • No interference problems with the environment • Estimate boundaries of objects	• Requires ambient light to illuminate the field of view • Susceptible to changes in light, dust, rain, and snow • High Computation cost • No depth information provided
Ultrasound	Sound waves above 20 kHz [emitted]	• Low cost, simple to operate • Lightweight, robustness, and fast response time • Good performance in poor lighting and transparent objects • Detect a wide range of materials	• Not suitable for medium to long distance range, normally more than 5 m • Affected by temperature, pressure, and ambient noise in the environment • Wide beam width and sensitivity to mirror-like surfaces cause specular reflections • Cannot distinguish shape and size • Must be perpendicular to the target as possible to receive the correct range data.
Infrared	Infrared Light [emitted]	• High-resolution, low-cost, and light weight • Faster response time than ultrasound • Can measure temperature	• Sensitive to weather conditions • Short detection range • Affected by dim light conditions

**Table 3:** A Summary Table of Obstacle Detection Sensor Features [Lidar: [10,50,58,59,60,61], Radar: [62,63], Camera: [64,65,66,67], Ultrasound: [68], Infrared: [69].

SENSORS	RANGE	MASS	OPERATING TEMPERATURE	FIELD OF VIEW	ACCURACY	RESOLUTION	DIMENSION	POWERS	COST	OUTPUT
3D LIDAR [Velodyne VLP 16] 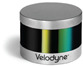	30–100 m	830 g	−10 °C to 60 °C	360° horizontal, 30 ° vertical	± 3 cm	Angular resolution: 2° vertical, 0.1–0.4° horizontal	103 mm diameter × 72 mm	Voltage: 9 v–18 V Power Consumption: 8 W	AUD 5000+	•Up to 300,000 points per second •100 Mbps Ethernet connection •UDP packets containing distances, calibrated reflectivities, rotation angles, synchronized time stamps (μs resolution)
2D LIDAR [RPLIDAR A2M8]	0.15–12 m	190 g	0 ℃ to 40 ℃	360°	1% of range (</= 3 m), 2% of range (3–5 m), 2.5% of range (above 5 m)	Angular: 0.45° Range: </= 1% of range (below 12), </= 2% of range (12 m ~16 m)	76 mm diameter × 41 mm	Voltage: 5 V Current: 450 mA–600 mA Power Consumption: 2.25–3 W	AUD 640	8000 points obtained with 10 Hz rotational speed
1D LIDAR [TF Mini Lidar]	0.3–12 m	6.1 g	−20°C to 60 °C	2.3°	1% (0.3 m–6 m), 2% (6 m–12 m)	5 mm	42 × 15 × 16 mm	Voltage: 4.5 V–6 V Power Consumption: 0.12 W	AUD 75.36	Distance obtained at 100 Hz
Solid State Lidar [ibeo LUX 4L] 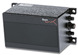	Up to 50 m	998.7 g	−40 °C to +85 °C	110° (H) × 3.2° (V)	10 cm	Angular Resolution (H × V): 0.25° × 0.8° Range Resolution: 4 cm	164.5 × 93.2 × 88 mm	Voltage: 9–27 V Power Consumption:7 W	-	Distance and echo pulse width
Radar [Delphi ESR] 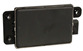	Long range: 1–174 m Mid-range: 1–60 m Velocity range: < −100 m/s to 40 m/s Lateral: −20 m/s to +20 m/s	575 g	-	Horizontal Long range: ±10° Midrange: ±45° Vertical: 4.2° to 4.75°	Range: < =/- 0.5 m (long), < ± 0.25 m (mid) Range rate: < ± 0.12 m/s Azimuth angle Centroid: < ± 0.3° (corner reflector targets in long-range),< ± 0.5°(other targets in long range), < ± 1.0° (mid-range)	Azimuth angle: <3.5° (long range) <12 ° (mid-range)	173.7 × 90.2 × 49.2 mm	-	AUD 2500	The estimated centroid of the detected object which includes the range to the centroid, its bearing angle, its longitudinal and lateral speeds, its acceleration, and the power of the returned signal (Φ).
Camera [Raspberry pi camera]	-	3 g	−30 °C to 70 °C	53.50 ± 0.13° (H), 41.41 ± 0.11° (V)	-	Still resolution: 5 MP Sensor resolution: 2592 × 1944 pixels	25 × 24 × 9 mm	Power Consumption: 325 mW	AUD 25	Images: 1080 p @ 30 fps, 720 p @ 60 fps, 480 p @ 90 fps
Stereo Camera [Roboreception RC Visard 160]	Depth Range: 0.5–3 m	850 g	0 °C to 50 ℃	Horizontal 61°, Vertical 48°	Depth Accuracy: 0.4–13 mm	Image resolution: 1280 × 960 pixels, 1.2 MP Lateral Resolution: 0.5–2.8 mm Depth Resolution: 0.1–3.3 mm	230 × 75 × 84 mm	Voltage: 18–30 V Power Consumption: 25 W	-	Right and left rectified image, depth image, confidence image at 0.8–25 Hz frames per second
TOF Camera [IFM O3D03]	3–8 m	766.95 g	−10 °C to +50 °C	60° × 45°	-	Image Resolution: 352 × 264 pixels	72 × 65 × 82.6 mm	Voltage: 20.4–28.8 V Current: <2400 mA Power consumption: 10 W	AUD 3000	3D image data obtained with a reading rate of 25 HZ
Ultrasound [HC SR04] 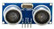	2 cm–4 m	8.5 g	−15 °C to 70 °C	30° conical	3 mm	-	45 × 20 × 15 mm	Voltage: 5 V DC Current: 15 mA	Under AUD 5	Distance estimated from the time between send (eight k40 Hz signals) and received pulses
Infrared [Sharp GP2Y0A02YK0F] 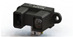	20–150 cm	4.8 g	−10 °C to 60 °C	10°	-	-	44.5 × 18.9 × 21.6 mm	Voltage: 4.4 V–5.5 V Current consumption: 33mA	AUD 30	Distance

**Table 4:** Selected obstacle detection sensors, type of wearables, sensor specifications, and feedback for assistive devices.

Obstacle Detection Sensor	Number of Sensors	Device	Type of Obstacle	Mass	Range	Feedback	Cost	Reference
Ultrasound	5	Wearable jacket	Path obstacles	3 g	2 cm–400 cm	Buzzer, Vibrator	Low	[82]
	2	Belt	Near object, distant object	Light	2 cm–400 cm	Vibration	Low	[92]
Camera	2	Bicycle Helmet	Foreground object	Light	10–20 m	Acoustic Feedback	Low	[81]
Microwave radar	1	Cane	Floor/suspended obstacles	Light	1 m–5 m	Acoustic, Vibration	Low	[93]
Infrared sensor	2	Foldable Stick	High-level /floor level obstacle, staircases	Light	Up to 200 cm	Audio through earphone	Low	[94]
ToF Distance sensor	7	Belt	Low and High Obstacle	8 g	0–14 m	Vibration belt	High	[83]
Tfmini Lidar and Ultrasound	1 each	Smart Glasses	Obstacles within the 1.7 m height, descending stairs	Light	10 cm–12 m (Lidar), 2 cm–300 cm (ultrasound)	Buzzer, Audio	Low	[79]
Lidar and Web Camera	1 each	Haptic Strap	Chair, person, bottle, bicycle	Light	*Not given*	Vibration and Audio	Low	[95]
Asus Xtion Pro camera	1	Strap on chest, Handcuff	Path Obstacles	Medium	0.8–3.5 m	Vibration	High	[96]
Microsoft Kinect	1	Strap on neck	Path Obstacles	Medium	*Not given*	Audio	Medium	[85]

**Table 5:** Main features of the obstacle detection shoe systems taken from the reviewed literature.

Shoe Name	Application	Sensors	Processor Board	Additional Device in Overall System	Additional Sensing	Accuracy	Alerting Technique	Battery Life	Detection Range
Smart-Alert Walker [91]	Visually Impaired	Ultrasound on shoes (obstacle), Camera on the cane (obstacle), Water Sensor (Moisture)	Arduino	Smart-fold cane	Water sensing	95.54% for common obstacles	Vibration Alert in leg, Audio output for the detected obstacles	-	-
Smart Bottine [32]	Autistic People	Ultrasonic sensor HC SR04 (obstacle) Infrared(insect) IMU MPU6050 (fall)	NodeMCU (ESP8266)	Smart Phone	Insect/Reptile Detection, Fall Detection, Location Tracking	-	Smartphone, Piezo buzzer	-	-
Smart Shoe [33]	Visually Impaired	Ultrasonic sensor HCSR04(obstacle) Infrared sensor(pothole) Moisture sensor (water) Temperature sensor LM35 (Fire)	Arduino Nano	Smart Phone	Location tracking, Pothole Detection, Hot object detection, Slippery Surface, health tracking	-	Voice sent to user’s headphone	3–4 Hrs	20 cm–4 m
Smart Shoe [34]	Visually Impaired	Ultrasonic Sensor HCSR04 (obstacle), Accelerometer ADXL335 (Foot motion)	STM32L432KC	Smartphone	Gait sensing, Fall detection	-	Vibration Motor	-	-
Smart Shoe system [36]	Visually Impaired	Ultrasonic Sensor, Fisheye camera	Raspberry pi Zero (streaming, actuation), Smartphone(detection)	Smart phone	Navigation	-	Vibration Motor	-	-
Shoe System [38,100]	Visually Impaired	Ultrasonic (obstacle), Infrared sensor (obstacle), Force sensitive resistor (Shoe wearing)	ARM cortex M3 LPC1768	-	Detect whether the shoe is worn by user	-	Buzzer	-	-
Real Time Assistive Shoe [35]	Visually Impaired	Ultrasonic sensor (obstacle), Moisture sensor (soil moisture)	Renesas RL78/G13	Smartphone	Moisture Detection, Navigation	-	Audio output	-	2 cm–80 cm
Clear Vision- Smart Shoes [101]	Visually Impaired	Ultrasound HCSR04	Arduino Nano (ATMEGA328)	Knee Band	Height Detection	-	Vibration	-	-
Smart Assistive shoe [102]	Visually Impaired	Ultrasound HCSR04	NodeMCU	Smartphone	Shoe Position Finder	-	Vibration	-	-
Smart Shoe [103]	Visually Impaired	Ultrasound	Arduino UNO	Smart glasses	-	-	Vibration	-	-
Smart Shoe [104]	Visually Impaired	Ultrasound HCSR04	Arduino Nano	-	-	-	Buzzer	-	-
Obstacle Detection Shoe [30]	Visually Impaired	Infrared sensor (obstacle, Accelerometer (Shoe direction)	Arduino 101 board	-	Shoe Direction	-	Piezoelectric Buzzer	-	-
Smart Shoe [98]	Visually Impaired	Ultrasound HCSR04, Water Sensor (wet), MPU6050 sensor(fall)	Arduino Mega	-	Wet detection, Fall detection	Overall accuracy-95.33%, Sensitivity of 98% and false detection rate of 5.3%.	Audible notification and vibration motors	2 h	2 cm–300 cm
COMPASS [105]	Visually Impaired	Ultrasound (obstacle), Raspberry Pi camera (text)	ESP32 development board(shoe) Raspberry Pi (bracelet)	Smart Bracelet, Smartphone	Text Detection	-	Beeper	-	-
Blind Shoe [37]	Visually Impaired	Ultrasonic sensor (obstacle), Water level sensor(water)	Arduino UNO	Smartphone	Slippery or wet surface	97.33%	Buzzer, Audio	-	2 cm–4 m, 15 degree
BUZZFEET [99]	Visually Impaired	Ultrasound (obstacle), Infrared (pit)	Arduino Lilypad	Audio processor module	Pit detection	-	Audio	-	-
NavGuide [31]	Visually Impaired	Ultrasound(obstacle), Liquid Detector Sensors (wet floor)	Customized microcontroller	-	Wet floor Detection	-	Audio, Vibration	850–1000 min	-
Fall Prevention Shoes	Elderly	Line laser (obstacle), Camera (obstacle, gait)	-	-	Gait detection	-	Alarm message	-	0.5–1 m
IPrevent Shoes [106,107]	Senior People	Radar	Laptop	-	-	-	-	-	-
Smart Shoes [108]	Visually challenged	Ultrasonic sensor	-	-	-	89.5%	Tapping at the foot arch	5 h	0–2 m (regular) 0–1 m (crowd)

**Table 6:** Microcontroller boards used for obstacle detection shoes. The features are taken from the papers and datasheets.

Microcontroller Board	Description	Reference
Arduino	Controls, processes, and generates all inputs and outputs. It receives the echo signals from the ultrasonic sensor that trigger it to take further actions and checks if the obstacle is there. It generates an immediate alert using a buzzer. It also generates a caption for the image captured by a camera and later converts that caption into speech that is played through an audio device.	[91]
	Arduino Nano—small, complete, breadboard-friendly board based on ATmega328 CPU clocked at 16 MHZ, 2 KB SRAM, 32 KB flash, 22 digital I/o pins, 8 analog pins and mini-USB port.	[33,101,104,114]
	Arduino UNO-equipped with the well-known ATmega328 P and the Atmega 16U2 Processor.	[37,103]
	Arduino 101 Board—To adjust the detection range of the sensor according to the walking direction, Arduino 101 boards, which contain Bluetooth, a six-axis accelerometer, and a gyrometer, were utilized.	[30]
	Arduino Mega—Atmega 2560-based with 54 digital input/output pins (of which 15 can be used as PWM outputs), 16 analog inputs, 4 UARTs (hardware serial ports), a 16 MHz crystal oscillator, a USB connection, a power jack, an ICSP header, and a reset button.	[98,115]
	Arduino Lilypad Atmega 328 V, 1 KB SRAM,512 bytes EEPROM, 8 MHZ clock speed, The Arduino Lilypad is attached with APR Module, GSM Module and Sensors. This Lilypad recognize the instructions sent by the Ultrasonic and IR sensors and works accordingly. It operates on 5v and is programmed by using Arduino IDE simulation platform. It receives and sends instructions accordingly.	[99]
NODE MCU	3.3 V operating voltage, 80 MHZ clock speed, 4 MB flash memory, 64 KB RAM, 11 digital pins, 1 analogue pin on this board and built-in Wi-Fi 802.11 b/g/n. The ESP8266 LX106 microcontroller on the NodeMCU receives data from attached sensors, process the data, and use the uploaded code such as SSID of the Wi- Fi network, the password for the Wi-Fi network, to communicate with smartphone.	[32,102]
STM32L432KC	Ultra-low-power microcontrollers based on the high-performance Arm^®^ Cortex^®^-M4 32-bit RISC core operating at a frequency of up to 80 MHz with floating point unit, 1.71 V to 3.6 V power supply.	[34,116]
Raspberry Pi Zero	The board incorporates a quad-core 64-bit Arm Cortex-A53 CPU, clocked at 1 GHz, 512 MB LPDDR2, 2.4 GHz 802.11 b/g/n wireless LAN and Bluetooth 4.2. The streaming of sensors data and actuation on shoe will be performed by the raspberry pi, while the obstacle detection is performed by smartphone.	[36,117]
ARM cortex M3 LPC1768	A flash memory of 512 KB, 64 KB data memory, a processor frequency of 100 Hz, 13 general purpose input-output (GPIO) registers _ 6 pulse width modulation (PWM) pins, 8 channel 12-bit analog to digital converter (ADC). The ultrasonic and infrared data given to the ARM cortex M3 microcontroller which determines if an obstacle is present or not.	[38]
Renesas microcontroller	Low level power consumption with supply voltage varying from 1.6–5.5 volts, the execution time can be varied from 32 Mhz–32 kHz, consists of 64 pins which include code flash memory, DMA controller, high-speed on-chip oscillator, serial interface, data flash memory.	[35]
ESP32 Development Board	ESP32 is a development board that incorporates both Wi-Fi and Bluetooth, which makes it a good choice to be utilized in projects related to embedded systems. It has Tensilica Xtensa Dual-Core 32-bit LX6 microprocessor which operates at either 160 or 240 MHz.	[105]

## Data Availability

Not applicable.
